# Assessment of Regional Ecosystem Health—A Case Study of the Golden Triangle of Southern Fujian Province, China

**DOI:** 10.3390/ijerph15040802

**Published:** 2018-04-19

**Authors:** Ziyan Wang, Lina Tang, Quanyi Qiu, Huaxiang Chen, Tong Wu, Guofan Shao

**Affiliations:** 1 Institute of Urban Environment, Chinese Academy of Sciences Urban Environment and Health Key Laboratory, Xiamen 361021, China; zywang@iue.ac.cn (Z.W.); qyqiu@iue.ac.cn (Q.Q.); hxchen@iue.ac.cn (H.C.); twu@iue.ac.cn (T.W.); geoshao@gmail.com (G.S.); 2 University of Chinese Academy of Sciences, Beijing 100049, China

**Keywords:** the Golden Triangle of Southern Fujian Province, pressure-state-response model, ecosystem health

## Abstract

Intensifying urbanization and rapid population growth in Fujian Province, China, has caused pollution of air and water resources; this has adversely impacted ecosystems and human health. China has recently begun pursuing a massive infrastructure and economic development strategy called the Belt and Road Initiative, which could potentially cause further environmental damage. Evaluations of ecosystem health are therefore a first step towards identifying the potential impacts from the development and planning sustainable development strategies in the Golden Triangle of Southern Fujian. To this end, our study analyzed landscape patterns and evaluated ecosystem health in this region. We used an index system method to develop a pressure–state–response (PSR) model for assessing the region’s ecosystem health. We found that: (1) the landscape type with the greatest area in the study region is cultivated land and there were no areas that were undisturbed by human activity; (2) the overall ecological health of the region is good, but there is distinct variation across the region. This study incorporates the landscape pattern into an evaluation of ecosystem health. Using counties as evaluation units, we provide a general evaluation index for this scale. The methods reported here can be used in complex ecological environments to inform sustainable management decisions.

## 1. Introduction

The Golden Triangle of Southern Fujian Province, which includes the cities of Xiamen, Zhangzhou, and Quanzhou in China, is a coastal ecosystem where urbanization has facilitated increased productivity and economic development [1]. However, the process of urbanization has also caused severe air pollution, soil erosion, ecosystem degradation, and biodiversity loss [2]. Pollution and the degradation of ecosystems threaten the region’s economic and social development [3,4]. In the face of a rapidly deteriorating ecological environment, it is important to evaluate whether these changes will harm human beings or cause environmental or economic crises [5], and to determine how to manage ecosystems. A rational and effective approach to evaluating the health of regional ecosystems can provide practical guidance for designing policies that achieve sustainable development [6].

As ecological threats continue to emerge, research on conducting ecosystem health assessments has gradually matured, leading to the development of different theories of ecosystem health [7]. In 1992, the WHO proposed the concept of “Healthy Cities”, which it defined as a combination of a healthy population, a healthy environment, and a healthy society. Healthy cities work to improve their environments, expand their resources, and enable urban residents to support each other to maximize their potential. In 1999, Rapport defined ecosystem health as, “the state, condition, or performance of an ecosystem defined by the suitable target standard” [8]. In other words, a healthy ecosystem should include two aspects: “one is the ability to meet the reasonable demands of human society, the other is the health and integrity of maintaining and updating the ecological environment and the ecosystem, as well as the health and social health of urban dwellers” [8]. In 1997, Colin wrote that healthy urban ecosystems not only promote the health and integrity of natural and artificial environments, but also promote the social and physical health of urban residents [9]. The conceptual framework for healthy cities defined by Hancock is based on the relationships between the economy, the environment, and society [10]. However, a research report by the International Development Research Centre emphasizes the importance of the relationships between social, economic, cultural, and political factors within the city and its surrounding environment [11]. Since 2000, Chinese scholars have paid increasing attention to research on ecosystem health and have improved our understanding of the concept. For example, Zeng et al. [12] and Ren et al. [13] completed a comparative analysis of ecosystem health concepts. In addition, a theoretical framework, connotation, index, method, and direction for ecological system health were introduced [14,15,16,17,18]. The most widely accepted view at present is that ecosystems are considered stable and sustainable when they remain healthy in the face of natural or human disturbances [16,17].

Ecological health assessment studies at provincial and national scales are relatively common in China [19], but research on smaller scales is rare, particularly those based on the landscape patterns. This paper uses the Golden Triangle of Southern Fujian as a study region to discuss the method for assessing ecosystem health, which may help inform ecological management decisions in the face of increasing urbanization. We used multidisciplinary methods, including the pressure—state—response (PSR) model, to assess the region’s ecological health. Using the PSR model, we revealed links between all elements of ecosystem health and established an indexing method to evaluate standards of ecological health.

## 2. Materials and Methods

There are two established methods for evaluating ecosystem health: (1) the indicator species method; (2) the index system method [15]. The indicator species method is mainly used for natural ecosystems, including forests, extreme environments, and wetlands, and is based on evaluations of indicator species, key species, and endemic species [19]. This method does not consider socio-economic and human activity factors, so it is not suitable for evaluating ecosystem health in regions experiencing intensive human activity.

The index system method incorporates the characteristics and service functions of an ecosystem and is therefore more comprehensive than the indicator species method. It not only employs indexes of ecosystem health, but also employs indexes of structure, function, process index, social economy, landscape pattern, and land use. This approach has an obvious advantage in evaluating ecosystem health in terms of its structure and function. The index system is currently the most commonly used method and can be applied in evaluations of watershed ecosystem health, regional ecosystem health, and global ecosystem health. Because it is difficult to find appropriate indicator species to assess ecosystem health at a regional scale, we used the index system method in this study.

Here, we conducted a two-part evaluation of ecosystem health in the Golden Triangle of Southern Fujian: (1) according to the basic theory of landscape ecology, we selected a suitable landscape pattern index to analyze the present landscape pattern in the study area; (2) using the PSR model, we established a system for evaluating ecosystem health. However, the main intention of this article is ecosystem health assessment. In this paper, the landscape pattern index is used as a basic index for the assessment of the regional ecosystem health.

### 2.1. Study Area

**Figure 1:**
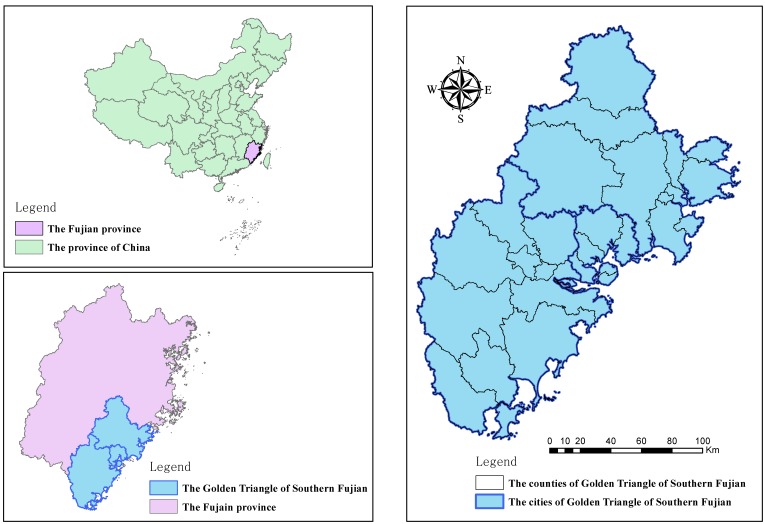
Location of the Golden Triangle of Southern Fujian Province.

The Golden Triangle of Southern Fujian Province covers the coastal region of China and includes the cities of Xiamen, Quanzhou, and Zhangzhou (Figure 1). The region is located at longitude 116°53′–119°05′ East, latitude 23°32′–25°56′ North and the area is 25,315.39 km^2^, which represents 1/5 of the total area of Fujian province. The region surrounding the Golden Triangle of Southern Fujian is mountainous and undulating. The terrain slopes upward from northwest to southeast. The influence of monsoon circulation and alpine topography is significant in the Golden Triangle of Southern Fujian. Regional average annual temperatures are 18–25 °C. Rainfall is very abundant, averaging 1500–2100 mm annually. Soil types in the region are complex and diverse and are classified into brick and red soil, clay soil, gravel soil, windblown sand soil, saline soil, tidal soil, and others. The total human population is greater than seventeen million people, accounting for about 40% of the population of Fujian Province. The region’s economic output accounts for 55% of the total output of Fujian province and it is one of the most economically dynamic regions in China.

### 2.2. Data Sources

This paper focuses on the evaluation of ecosystem health in Southern Fujian. The data sources used in the study include the following: (1) land use data for 2015 were based on Landsat TM (TM, Thematic Mapper) images (USGS/NASA, United States Geological Survey/National Aeronautics and Space Administration, https://eros.usgs.gov/about-us/data-citation) and were provided by the Data Center for Resources and Environmental Sciences of the Chinese Academy of Science; (2) the 2015 “Statistical Yearbook of China”, published by the National Bureau of Statistics of China, which provides data on population, comprehensive economy, industry, transportation, trade, foreign trade, fixed asset investment, education, culture, health, people’s livelihood, social security, municipal utilities, and environmental protection; (3) data on soil erosion control rates, industrial waste water treatment rates, utilization of industrial solid waste, and other environmental protection data, provided by the State Environmental Protection Agency (SEPA) for 2015; (4) economic data provided by the Xiamen Municipal Bureau of Statistics, the Quanzhou Municipal Bureau of Statistics, and the Zhangzhou Municipal Bureau of Statistics.

### 2.3. Analysis of Landscape Pattern

Appropriate landscape indexes should be selected based on their characteristics, purposes, and contents to achieve accurate, quantitative descriptions of the research objects [20]. Many researchers have pointed out that indexes should be chosen based on their ability to explain the specific research question being studied [21,22,23]. From the visual inspection in this study, it was clear that the number of landscape patches was quite high in the research areas. If we had calculated the landscape index of each patch, the number of required calculations would have been extremely large and the results would not have produced a significant analysis of the landscape pattern. Therefore, this study calculated the landscape index at the landscape level to analyze the landscape pattern of the research area.

This study uses Landsat TM remote sensing images as the main data source in addition to data from the “Fujian Land Renewal Survey Database” and the “Statistical Yearbook”. The interpretation of the remote sensing images was carried out according to the classification of land use status (GB/T21010-2007). Land use types were classified into woodland, agricultural land, grassland, and construction land. The interpreted land use data was then used to calculate landscape patterns in ArcGIS 10.3, which is developed by Esri (Esri, Environmental Systems Research Institute, Inc., Redlands, CA, USA) in the USA.

#### 2.3.1. Shannon’s Diversity Index

The Shannon diversity index is used to measure the complexity of ecosystem structure. The complexity of landscape composition is determined primarily by the number of elements in the landscape and their proportion in the landscape. The Equation (1) for calculating the Shannon diversity index (*H*) is as follows:

(1) $$H=-\overset{n}{\underset{i=1}{\sum }}P_{i}ln\left(P_{i}\right)$$

where $P_{i}$ refers to the probability of the emergence of a patch *i* in the landscape, and *n* refers to the total number of patch types in the landscape.

*H* is always ≥0. When there is only one type of patch in the landscape, *H* = 0, indicating that the landscape has no diversity and is uniform. When the number of patch types increases or the proportion of each type of patch is similar, the value of the diversity index increases. *H* indicates the degree of diversity of landscape types, not species diversity. Our calculations revealed that landscape patch complexity was proportional to landscape area.

#### 2.3.2. Shannon’s Evenness Index (SHEI)

The Shannon’s evenness index (SHEI) reflects the uniformity of the distribution of different landscape types in a region. It is usually represented by the ratio of *H* to its maximum value. Equation (2) for calculating SHEI is as follows:

(2) $$\mathrm{SHEI}=\frac{H}{H_{max}}=\frac{-\sum_{i=1}^{n}P_{i}\ln\left(P_{i}\right)}{\ln\left(n\right)}$$

where H is Shannon’s diversity index, and $H_{max}$ is the maximum Shannon’s diversity index. The uniformity of landscape type distribution is greatest when SHEI = 1.

#### 2.3.3. Landscape Fragmentation Index (FN)

The landscape fragmentation index refers to the degree of fragmentation of the landscape. It can reflect the intensity of human disturbance. It is an important index of landscape heterogeneity, which is positively correlated with species diversity. Equation (3) for calculating FN is as follows:

(3) $$\mathrm{FN}=\frac{N}{A}$$

where *N* is the total number of regional landscape patches, *A* is the total area of the regional landscape (km^2^).

The lower the value of FN, the less fragmented the landscape. The higher the value of FN, the more fragmented the landscape.

### 2.4. Evaluation Indexes Used in the PSR Model and Determination of Index Weight

#### 2.4.1. PSR Model

The Organisation for Economic Co-operation and Development (OECD) and the United Nations Environment Programme (UNEP) jointly proposed the PSR model for evaluating the causal relationships of ecological environmental indexes [24]. The model is based on causality, that is, the specific human activities that exert pressure on the environment, causing changes in its original function or the number of natural resources it provides (i.e., state). Human society takes certain measures to respond to these changes in order to restore the environmental quality or prevent environmental degradation (i.e., response) [25,26,27]. The factors pressure, state, and response influence each other and the PSR model reflects this, highlighting the causality between pressure on the environment and environmental degradation. The PSR model is the whole process of making decisions and formulating countermeasures. The best evaluation of ecosystem health incorporates the interactions between human activity and the ecosystem. This study not only considers the influence of current human activity on the ecosystem, but also considers how human activity is influenced by societal structure and the economy. We therefore constructed a comprehensive index for evaluating regional ecosystem health that is based on the PSR model. The PSR model organizes environmental indexes according to their interactions and influences on human and environmental systems [28].

#### 2.4.2. Evaluation of Indexes

Within the PSR framework, environmental issues can be expressed using three different but interlinked types of indexes. Pressure indexes represent the causes of environmental deterioration. State indexes are used to measure changes in the ecosystem caused by human behavior. Response indexes represent the efforts that society is making to reduce environmental pollution and damage to resources [26]. In order to analyze the problems in a comprehensive and systematic way, we must consider a number of factors in the ecological health assessment. Each index reflects a portion of the information available on ecological health and does so in a slightly different way. However, the massive indexes increase the amount of computation and the complexity of analysis; therefore, it is necessary to select appropriate indexes. Principal component analysis is an ideal tool for solving this problem using IBM SPSS (IBM SPSS, International Business Machines Corporation Statistical Product and Service Solutions, Armonk, NY, USA) Statistics 21.0, which was developed by Norman H. Nie et al. and was purchased by IBM in the USA.

Using the concept of dimensionality reduction, multiple indexes are converted into fewer comprehensive indexes, based on the premise of minimizing loss of information. Generally, the comprehensive index of transformation generation is the primary component, in which each principal component is a linear combination of the original indexes. Since each principal component is not related to any other, this improves the performance of the principal component rather than the original index. So, we can consider a select few principal components without losing too much information. Thus, it is easier to grasp the main contradictions and to reveal the regularity between the internal variables of things and improve analytical efficiency by simplifying the problem. The specific operational steps are as follows: (1) the initial analysis indexes are selected according to the research problem and PSR model; (2) finding the contribution rate of the eigenvalue and the cumulative variance, the number of the principal components and the correlation coefficient matrix of the original variable and the principal component; (3) calculating the ranking of the main components; (4) principal component indexes are added or deleted depending on the actual environment of the study region.

#### 2.4.3. Determination of Index Weight

The weight of each index has a significant impact on the evaluation results because the role of each index is not equal. In order to reflect the degree of importance of each index in the index evaluation system, we assign each index different weights.

Index weights can be determined using one of two methods: subjective empowerment and objective empowerment. In the subjective empowerment method, weights are decided based on the experts’ experience, using the Analytic Hierarchy Process (AHP), Delphi, and Palaeo decision-making methods [26,27,28]. In the mid 1970s, AHP was formally proposed by the American operational research scientist Thomas Saaty. It uses multiple schemes or multiple targets. The main feature is that it rationally combines qualitative and quantitative decision-making [29]. Delphi, first proposed by Helm and Dalke, has been applied in many areas of decision-making; it incorporates anonymous expert decisions that must formulate their decisions without conferring with other experts. After repeated consultation, induction, and modification, a consensus expert opinion is finally compiled and used as a basis for decision-making [30,31]. In the objective empowerment method, weights are calculated using statistical methods, including the weighted index method, the entropy weight method, and the mean square method [32,33,34]. However, each weight is influenced by the value of the specific evaluation index, which makes it difficult to show the relative importance of the evaluation indexes.

A large number of indexes mean that we have to construct a deeper, more quantitative, larger scale judgment matrix. As a result, the two comparisons of the analytic hierarchy process are 1–9; this explains its relative importance [35]. When we construct a judgment matrix, we are in accordance with the following criteria:

$a_{ij}=1$, Element i and element *j* are of the same importance to the upper level
$a_{ij}=3$, Element i is slightly more important than element *j*
$a_{ij}=5$, Element i is more important than element *j*
$a_{ij}=7$, Element i is much more important than element *j*
$a_{ij}=9$, Element i is of even greater importance than element *j*
$a_{ij}=2n$, *n* = 1, 2, 3, 4… The importance of elements *i* and *j* are between $a_{ij}=2n-1$ and $a_{ij}=2n+1$
  $$a_{ji}=\frac{1}{a_{ij}}$$

In this study, we used a scoring method to determine index weights according to the actual situation in the research areas. Although it is subjective, the subjective empowerment method is not random and therefore has scientific rationality because it can reveal the relative importance of the different evaluation indexes. Indexes with positive influences on the environment are represented with “+”; the greater the index weight, the greater its positive influence on the ecosystem. Indexes with negative influences on the environment are represented with “−”; the smaller the index weight, the more its negative influence on the ecosystem. In Table 1, C1 refers to the first index (i.e., land reclamation), and C2 refers to the second index (i.e., human interference index), and so on.

### 2.5. Evaluation Method

#### 2.5.1. Standardization of Indexes

The types of evaluation indexes are diverse and there are large differences between evaluation units. Evaluation indexes cannot be directly calculated by weighting. The quality of each index cannot be clearly defined, so it is difficult to directly compare the actual values of the indexes. The indexes must therefore be standardized to produce a unity of dimension before they can be synthesized [7]. Ecosystem health is a concept with a clear connotation but an unclear definition because the definitions of “healthy” and “unhealthy” have no clear definitions. 

In this study, the data were transformed using the extreme standardization method and the standardization value of the evaluation index was set between 0 and 10. For the evaluation index of positive ecological environment, it was necessary to make it have a larger standard value. For the evaluation index of negative ecological environment, it was necessary to make it have a smaller standard value. These two types of indexes used different equations when they were standardized.

The indexes with positive ecological impacts were standardized using Equation (4) [36]:

(4) $$A_{ij}=10\left(X_{ij}-X_{jmin}\right)/\left(X_{jmax}-X_{jmin}\right)$$

The indexes with negative ecological impacts were standardized using Equation (5) [36]:

(5) $$A_{ij}=10\left(X_{jmax}-X_{ij}\right)/\left(X_{jmax}-X_{jmin}\right)$$

#### 2.5.2. Comprehensive Evaluation of Ecosystem Health

To produce comprehensive index values for each evaluation unit, we used the comprehensive index evaluation method to weight the ecological health pressure, state, and response indexes. Equation (6) was used to calculate the comprehensive index values as follows:

(6) $$\mathrm{EH}=\overset{n}{\underset{i}{\sum }}W_{i}X_{i}$$

where EH is the ecosystem health value of each study region, $W_{i}$ is the weight of *i*, $X_{i}$ is the numeric value after index standardization, and *n* is the number of evaluation indexes.

Establishing an ecosystem health standard is key to assessing the health of the regional ecosystem. Standards of ecosystem health are based on human activity and depend on human judgment, which is often based on social interests, to determine the status of ecosystem health. The academic community has not yet agreed on an index system to use when assessing the health of regional ecosystems or the standard values of the indexes. Therefore, Table 2 includes the authors’ own values, which were developed from domestic and foreign indexes, city construction standards, expert opinions, and the eco-city index developed by China’s Ministry of Environmental Protection, which represent the recommended standards of ecosystem health [36,37,38,39]. On the basis of the existing literature, the author graded the evaluation value of ecological health. These existing studies have been verified with concrete examples and have a considerable degree of credibility. The ecosystem health status of the research area is divided into five grades: very good, good, normal, poor, and extremely poor. The comprehensive score and meaning of each level are listed in Table 2.

## 3. Results

### 3.1. Landscape Pattern Analysis

Within our study area, cultivated land is the landscape type that comprises the largest area. Human activity strongly influences the creation of different landscape types. The results of the landscape pattern index are shown in Table 3.

#### 3.1.1. Shannon’s Diversity Index (*H*)

**Figure 2:**
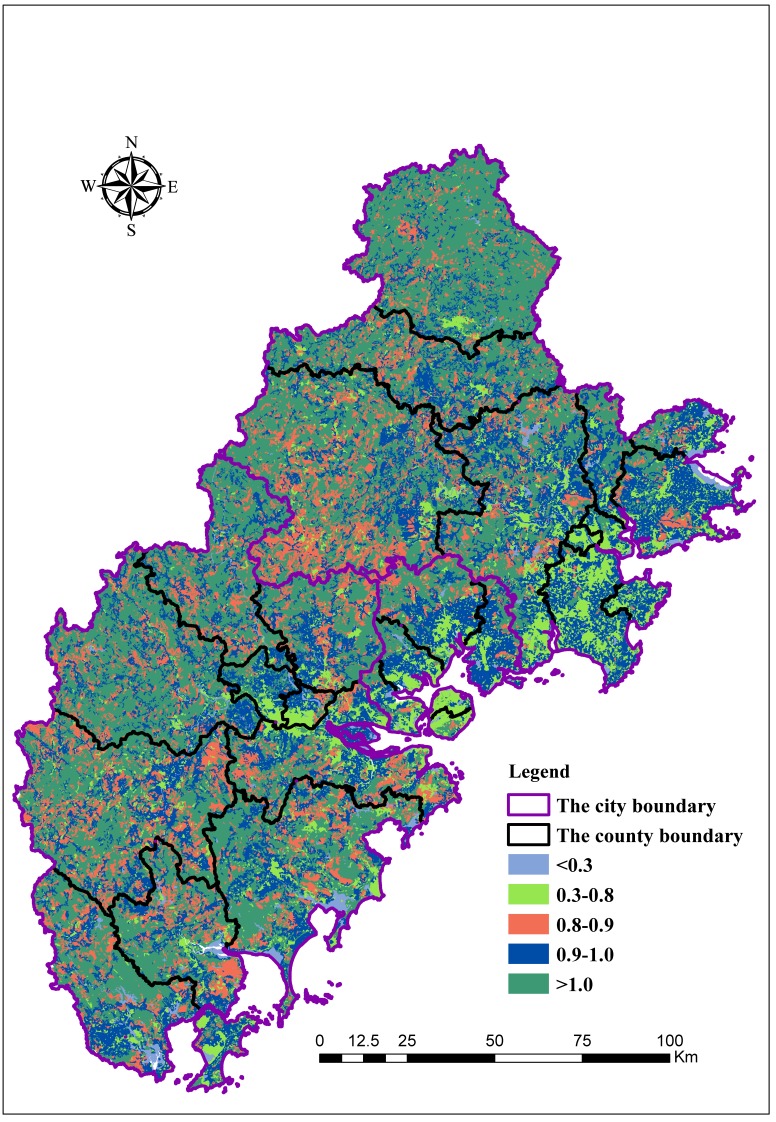
Evaluation results for Shannon’s diversity index (*H*).

When the distribution of landscape types is balanced, the value of *H* is high. As shown in Figure 2, the landscape diversity index value of the entire region is 0.78 and ranges between 0.42 and 1.23, depending on the county. The highest value is 1.23 in Dehua, which suggests that the diversity of various landscape types in this county is relatively balanced and the composition of landscape structure is more complex than in other counties. The landscape diversity of Si’ming is lowest, which is likely because of the high proportion of cultivated land (68.04%) and the low proportion of other landscape types.

#### 3.1.2. Shannon’s Evenness Index (SHEI)

**Figure 3:**
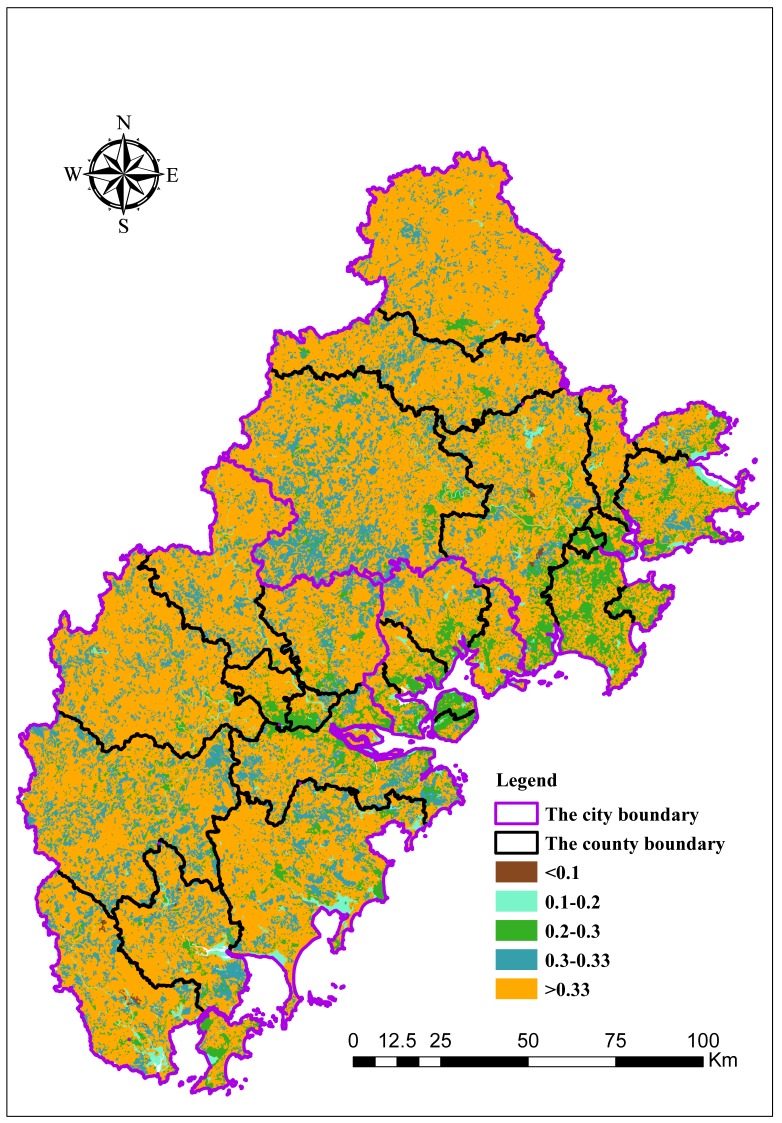
Evaluation results for Shannon’s evenness index (SHEI).

SHEI represents the degree of uniformity of landscape type distribution in a region. The greater its value, the more balanced the distribution of regional landscape types. As shown in Figure 3, the value of SHEI across the entire study area is 0.25. The order of the SHEI value, which from large to small, is consistent with the *H* value in all of the studied counties.

#### 3.1.3. FN

**Figure 4:**
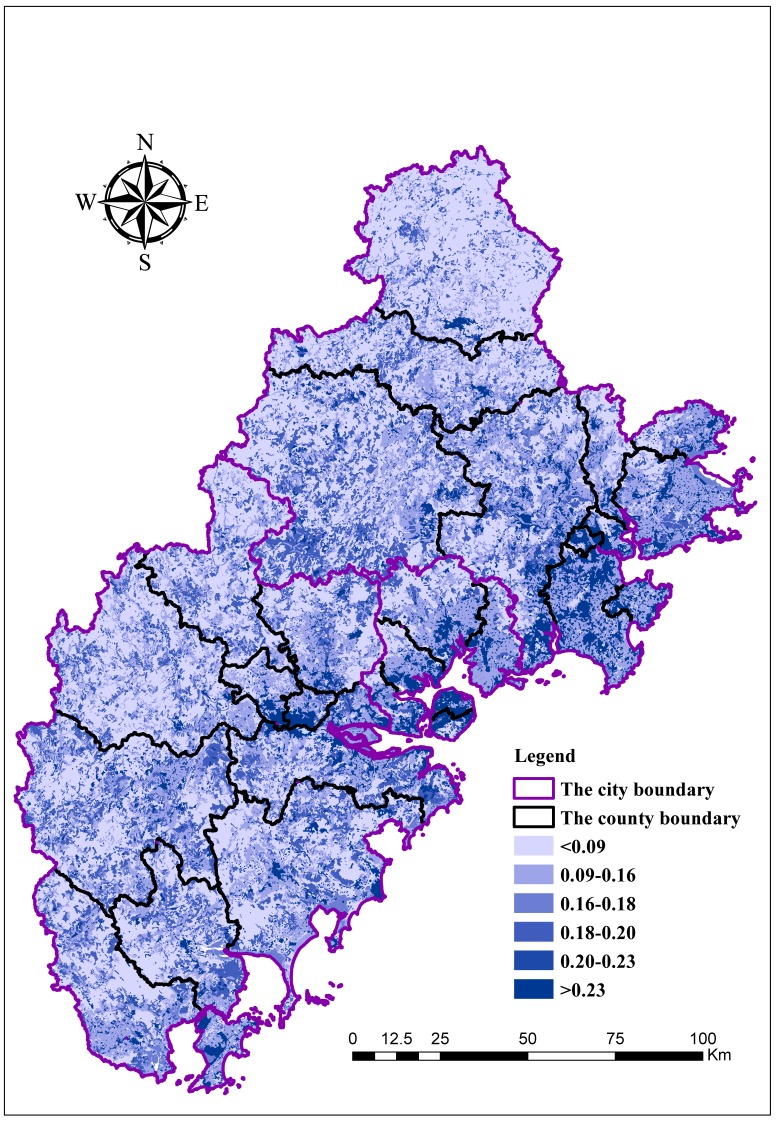
The evaluation results for landscape fragmentation index (FN).

As shown in Figure 4, the overall FN of the region is 0.15/km^2^. A high FN value indicates a high degree of landscape fragmentation.

### 3.2. Evaluation of Ecosystem Health

**Figure 5:**
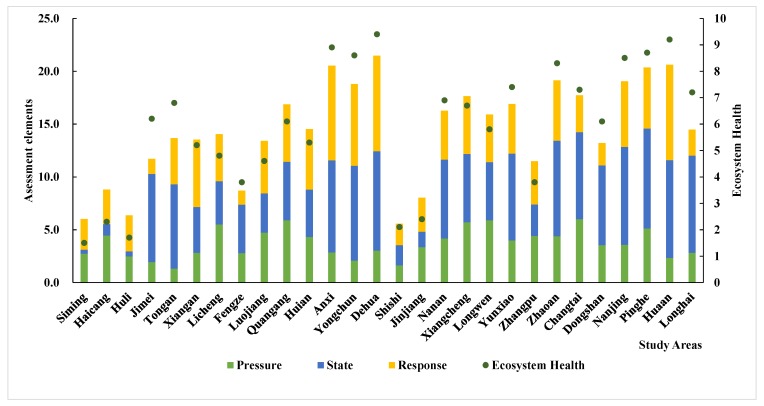
Ecosystem health in different counties.

**Figure 6:**
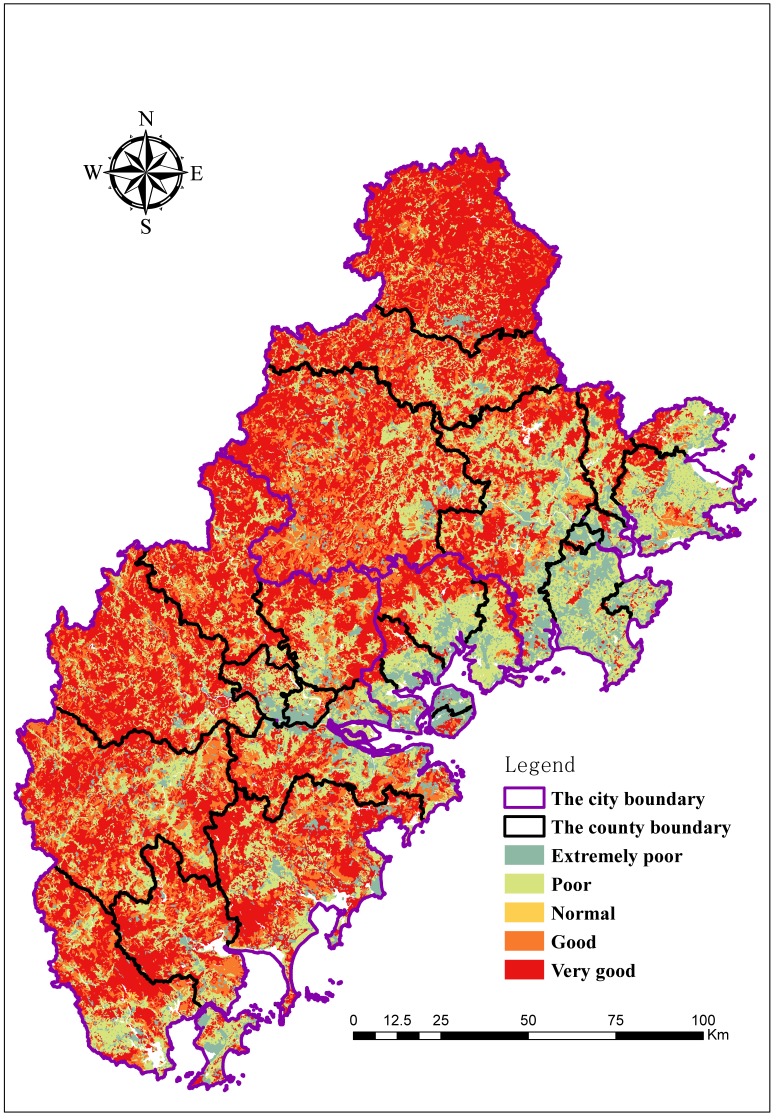
Ecosystem health in the Golden Triangle of Southern Fujian Province.

There are distinct variations in ecosystem health across the Golden Triangle of Southern Fujian. The ecological health throughout most of the study regions is good, but there is spatial heterogeneity, which follows a clear spatial distribution pattern. In general, the western region is superior to the eastern region. The level of ecosystem health in the west is better than in the east. As shown in Figure 5 and Figure 6, Anxi, Yongchun, Dehua, Nanjing, Yunxiao, and Hua’an are in good health, likely because these areas are relatively far from industrial areas, the effects of human activity are relatively small, and forest coverage is high. The vitality, restoring force, and service functions of the regional ecosystem are relatively strong in the counties mentioned above. The ecosystem health of Si’ming, Haicang, Huli, Licheng, and Shishi is poor, likely because of a high population density, a high level of human activity, and the irrational structure of the ecosystem.

The comprehensive evaluation of ecological health in different counties can be seen more clearly in Figure 5. Here, we can see that pressure, state, and response vary by region. The “pressure” of Anxi, Yongchun, Nan’an, Dehua, and Hua’an is small, while the “state” and “response” values are large. However, Shannon’s diversity index, Shannon’s evenness index, and the woodland coverage index are higher in these regions. These regions possess numerous enterprises and relatively developed economies, so the “state” and “response” values are higher. In Jimei, Tongan, Fengze, Longhai, Dongshan, and Changtai, the “pressure” and “state” values are high and the “response” value is relatively low. The primary reason for this result is that the level of economic development in these regions is low. Although the health of the ecosystem is relatively good based on the evaluation units, the economy should be actively developed in order to protect the natural environment. In the regions of Si’ming, Haicang, Huli, Shishi, Jinjiang, and Zhangpu, the “pressure” and “response” values are relatively high, but their “state” values are small. The overall ecological condition of these regions is relatively poor, primarily because these regions are more developed and there is more pressure on the environment. However, the infrastructure in these regions is high quality, which can reduce the negative impact of human activities on the environment. As a result, the response value of the ecosystem is high.

### 3.3. Analysis of Regional Ecosystem Health Elements in the Study Areas

**Figure 7:**
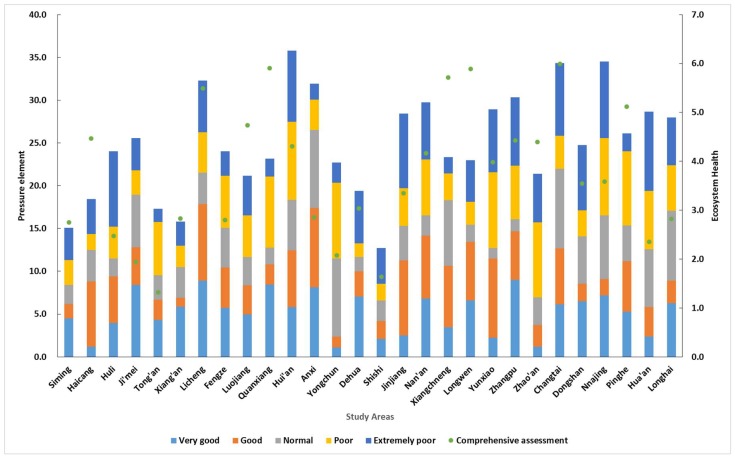
Analysis of the pressure elements in the study areas.

**Figure 8:**
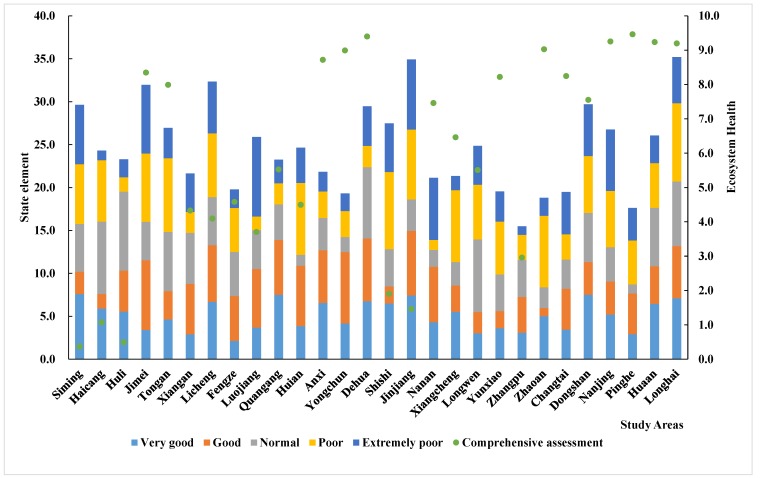
Analysis of the state elements in the study areas.

**Figure 9:**
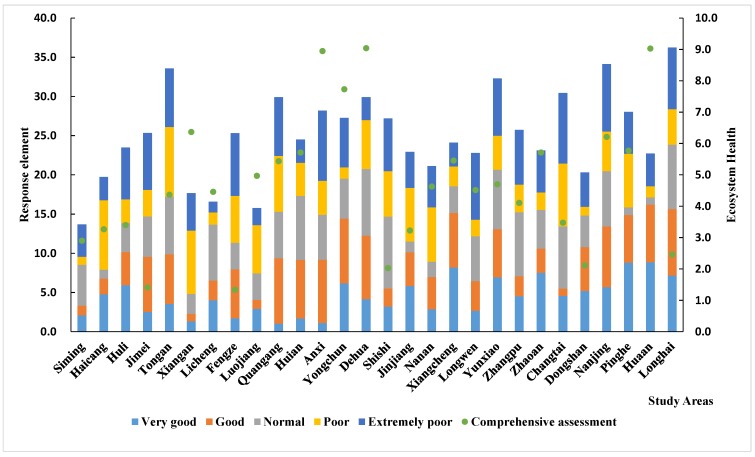
Analysis of the response elements in the study areas.

On the basis of Section 3.2, here the author further analyzes the impact of “pressure” “state” and “response” on the ecosystem health value. The PSR evaluation for the 28 counties is shown in Figure 7, Figure 8 and Figure 9. These average values are the result of a comprehensive ecosystem evaluation in the Golden Triangle of Southern Fujian regional ecosystem. (The main ordinate is the value of the stacked column and the longitudinal ordinate is the line diagram value).

#### 3.3.1. Pressure

The “pressure” element indicates the cause of the deterioration to the ecological environment. The greater the pressure, the worse the health of the ecosystem. We compared the pressure elements of 28 counties in different ecosystem health areas. The results are shown in Figure 7. The pressure elements are greatest in the Si’ming, Huli, Hua’an, Ji’mei, Shishi regions, where the regional GDP and foreign trade volume index are higher than those in other regions, and the development of regional economy is fastest.

#### 3.3.2. State

The “state” element is used to represent changes in the ecosystem caused by human interference with the natural environment. The developing economy in the region has led to the construction of convenient transportation and a high living standard in the population, which in turn has caused increased urbanization, overpopulation, and human disturbance to the ecosystem. We compared the state elements in 28 counties (Figure 8). The state elements are extremely poor in the Si’ming and Haicang regions, but are relatively good in the Changtai, Pinghe, Dehua, Nanjing, and Hua’an regions. 

#### 3.3.3. Response

The “response” element shows the institutional mechanisms established by society that are mitigating environmental pollution and resource destruction. We compared the response elements of 28 counties (Figure 9). The response elements are extremely poor in the Dehua, Hua’an, and Anxi regions. In recent years, the government has actively adjusted policies controlling the industrial structure in order to manage regional ecosystems more sustainably.

## 4. Discussion

In our analysis, the ecosystem health of counties within the research area showed a spatial difference, and also some regularity. What caused them and what did they mean? What impacts would they have? Was there is a relationship between urbanization and the health of the ecosystem? How can we adjust the process of regional development to ensure the ecosystem’s health? We will discuss and solve these questions in this section.

### 4.1. Identification of Main Controlling Factors

Ecosystem health is determined by the interactions between numerous ecological factors [41]. In order to determine the influence of different factors operating in different ecosystems, it is necessary to identify the main factors contributing to the regional ecosystem health [42]. Using a system that ranks ecosystems based on their deviation from a normal, healthy condition, we identified the main factors controlling ecological health (Equation (7)). On the basis of an average of 17 ecological factors influencing the ecosystem health in different regions, we calculated the relative deviation of different ecological indexes (the index numbers are ordered based on Table 1).

(7) $$F=\frac{X_{i}-\bar{X}}{\bar{X}}*100\%$$

where *F* is relative deviation, which refers to the main controlling factor; $X_{i}$ is the average value of the element i in one level of ecosystem health; $\bar{X}$ is the mean value of element i.

**Figure 10:**
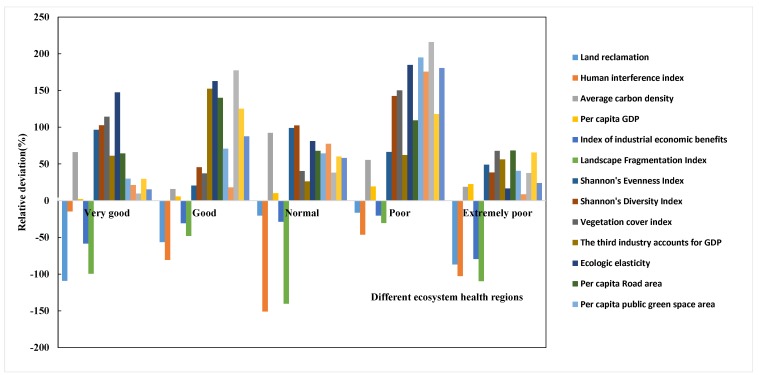
Relative deviation of the 17 factors in different ecosystem health regions.

In the Section 3.2, the authors analyzed the value of the ecological health assessment and the “pressure, state and response” of each region in the study area according to Figure 5 and Figure 6. We further divide the factors affecting the level of each ecological health grade in Table 4 and Figure 10. (C1–C17 refer to the index order from top to bottom in Table 1.)

Very good: in these areas, the main controlling factors are land reclamation, the vegetation cover index, ecologic elasticity, and per capita GDP. The levels of economic development and human interference are low. Although the land reclamation rate is high, ecosystem health is still the highest in these regions. In order to protect the natural environment, steps should be taken to actively develop the economy, improve income levels, improve the profitability of enterprises, and encourage sustainable development in these regions.

Good: in these areas, the main controlling factors are comprehensive industrial economic benefits, ecologic elasticity, per capita road area, and the treatment rate of industrial wastewater. Rapid economic development has led to a large population density in these areas. If there is irrational human activity that puts pressure on the regional ecosystem, the system is likely to slide quickly into a poor and then extremely poor state. Appropriate ecosystem management programs, which eliminate the limiting factors that affect the health of the ecosystem, will help the ecosystem develop in a healthy and orderly way.

Normal: in these areas, the main controlling factors are the vegetation cover index, ecologic elasticity, the utilization of industrial solid waste, and the average carbon density. In general, although the landscape diversity is high and the land reclamation rate is low, the ecosystem health is relatively normal.

Poor: in these areas, the main controlling factors are SHEI, water and soil conservation, *H*, and the area of per capita public green space. The SHEI and *H* values are high in these areas because the economy is relatively developed and values for “state” and “response” are high. In these regions, we suggest controlling population growth and land use intensity.

Extremely poor: in these areas, the main controlling factors are the vegetation cover index, ecologic elasticity, land reclamation, the human interference index, and FN. These regions have a low area of forested land and the vitality and elasticity of the ecosystem are very low. The irrational spatial structure of the landscape affects ecosystem services and the ability of the socioeconomic system to respond to changes in the environment. These areas should focus on environmental protection and increasing the coverage of forested lands.

### 4.2. Spatial Correlation between Ecological Health and Urbanization

**Figure 11:**
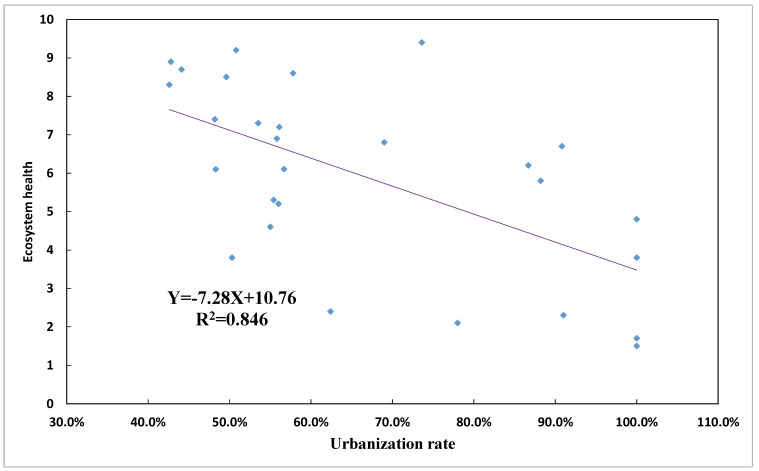
Relationship between ecosystem health and urbanization rate.

In order to verify the relationships between rapid urbanization and ecological health, we conducted regression analyses on values for the urbanization rate and the ecosystem health in the 28 counties. As shown in Figure 11, urbanization factors display a significant negative correlation with ecological health, at a significance level of 0.01. The higher the urbanization of the regional ecosystem, the lower the level of ecosystem health.

### 4.3. Sensitivity of the Indexes Used to Evaluate Ecological Health

The sensitivities of the ecological health indexes to evaluate ecosystem health were investigated.

**Figure 12:**
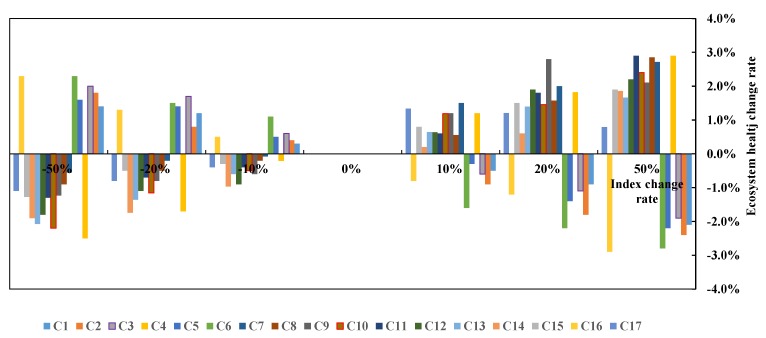
Ecological health index sensitivity.

When the evaluation index changes rated at ±10%, ±20%, ±50%, we analyzed how the ecosystem health evaluation value changed. This allowed us to identify the importance of each evaluation index to the overall ecological health level of the region and provide a framework for prioritizing projects that may impact the environment. The sensitivity analysis is shown in Figure 12.

Figure 12 shows that ecological health is sensitive to different indexes. Some indexes have a negative impact on ecological health, such as C1, C2, C3, C5, C6, and C16. Other indexes have a positive impact on ecological health. This analysis can provide a theoretical basis for the management of ecological health. Some management decisions may reduce the impact of negative indexes, for example, by integrating different land use needs and improving land use efficiency. This, in turn, reduces population density.

### 4.4. Innovation and Prospect

#### 4.4.1. Innovation Compared with Other Authors’ Previous Works

A robust theoretical system and research framework for assessing regional ecosystem health has not yet been developed. Therefore, this study attempted to explore the following:

At present, ecosystem health assessments and studies on the integrated development of the economy and the environment focus on cities, but largely ignore surrounding areas. However, efforts to achieve sustainable development in a region must be based on health assessments of a broader region. Here, we assess ecosystem health at the county level in order to gain an understanding of ways to balance economic development and ecosystem health. Using a PSR model, we built an evaluation system for ecosystem health at the county scale, and we correlated ecosystem health level with the degree of economic and social development. At the same time, we identified the main factors influencing ecological health in order to provide a basis for management decisions aimed at balancing economic development with ecological protection. This paper takes the landscape pattern into consideration for the ecosystem health evaluation. In our method for assessing ecosystem health, we began by selecting indexes, establishing an ecosystem health standard, and further improving the evaluation framework and index system. We also considered the influence of the landscape pattern and the relationship between ecosystems and urbanization.

#### 4.4.2. Future Work Prospect

Because of the imperfections of the data in the study area, this paper did not carry out a long-term dynamic assessment of the regional ecosystem health. Only a current regional ecosystem health assessment was carried out, and a long-term dynamic change series will be studied in the future.

The purpose of an ecosystem health assessment is to help formulate policies to deal with uncertain factors in the future. Therefore, the necessary situational analysis will be the focus of our next work. That is to say, assuming that a certain phenomenon or trend will continue in the future, we can predict the possible situation or consequences affecting the ecosystem.

Since the evaluation is based on relative standards, we should further study the evaluation criteria in order to better reflect the health of the regional ecosystem and further improve the research of this subject.

## 5. Conclusions

We discuss the theory and method of landscape pattern analysis and regional ecosystem health evaluation in the study region. Ecosystem health evaluation was conducted using a PSR model. We used the resulting landscape pattern analysis and ecosystem health evaluation system to carry out a quantitative and hierarchical study of ecosystem health. Local managers can use this protocol to inform management decisions that achieve more efficient use of resources while protecting ecosystems. The conclusions of this study are as follows:

(1) There is a wide variation in the ecosystem health of different cities; this is primarily due to the extremely low ecosystem health status of Xiamen compared with Zhangzhou and Quanzhou. The poor ecosystem health of Xiamen is primarily due to its development as the political, economic, and cultural center of Fujian. In Xiamen, economic development, urban infrastructure, and environmental facilities are superior to those in in Zhangzhou and Quanzhou.
(2) Ecosystem health is determined by the interactions between numerous ecological factors. In order to understand the influence of these factors on the ecological health of the area, we calculated the main factors affecting ecosystem health. Ecosystem health states were calculated using the mean values of the primary ecological factors influencing the ecosystem and by determining the main control factors in different ecosystems.
(3) Ecological health exhibits a significant negative correlation with the urbanization rate, GDP, and development in Southern Fujian. This suggests that urbanization and industrialization contribute to a decline in the ecosystem health.
(4) Because of the gaps in the available data, we were unable to perform a longitudinal study assessing changes in ecosystem health in the study region over time. We will attempt to address this issue in future research.

## Figures and Tables

**Table 1:** Evaluation of indexes using the pressure—state—response (PSR) model and determination of index weight.

Elements	Index Type	Index Name	Weight [36,37,38,39,40]
Pressure	Land	Land reclamation (−) C1	0.0678
	Population	Human interference index (−) C2	0.0661
State	Vitality	Average carbon density (−) C3	0.0501
		Per capita GDP (+) C4	0.0596
		Comprehensive industrial economic benefits (−) C5	0.0617
	Organization structure	Landscape fragmentation index (−) C6	0.0612
		Shannon‘s evenness index (+) C7	0.0665
		Shannon‘s diversity index (+) C8	0.0625
		Vegetation cover index (+) C9	0.0549
		Third industry accounts for GDP (+) C10	0.0631
	Restoring force	Ecological elasticity (+) C11	0.0674
	Support power	Per capita road area (+) C12	0.0631
		Per capita public green space area (+) C13	0.0516
Response	Natural response	Water and soil conservation (+) C14	0.0524
	Social and economic response	Treatment rate of industrial waste waste (+) C15	0.0553
		Utilization of industrial solid waste (−) C16	0.0543
		Environmental investment accounts for GDP (+) C17	0.0424

**Table 2:** Regional ecosystem health grade standard [36].

Health Levels	Health State	Assessment Value
1	Very good	8.0–10.0
2	Good	6.0–8.0
3	Normal	4.0–6.0
4	Poor	4.0–6.0
5	Extremely poor	0.0–2.0

**Table 3:** Landscape index values in different regions.

Study Areas	SHEI	H	FN
Si’ming	0.09	0.42	0.26
Haicang	0.18	0.68	0.18
Huli	0.12	0.53	0.23
Ji’mei	0.28	0.71	0.18
Tong’an	0.29	0.86	0.20
Xiang’an	0.26	0.72	0.18
Licheng	0.24	0.61	0.22
Fengze	0.19	0.58	0.24
Luojiang	0.25	0.62	0.22
Quangang	0.27	0.72	0.19
Hui’an	0.16	0.63	0.21
Anxi	0.30	0.83	0.19
Yongchun	0.31	0.98	0.17
Dehua	0.38	1.23	0.05
Shishi	0.21	0.69	0.11
Jinjiang	0.17	0.62	0.13
Nan’an	0.28	0.86	0.09
Xiangcheng	0.26	0.84	0.10
Longwen	0.18	0.78	0.16
Yunxiao	0.28	0.92	0.09
Zhangpu	0.29	0.96	0.08
Zhao’an	0.25	0.91	0.09
Changtai	0.24	0.81	0.12
Dongshan	0.27	0.88	0.07
Nanjing	0.36	1.08	0.04
Pinghe	0.31	1.02	0.08
Hua’an	0.33	1.06	0.07
Longhai	0.26	0.83	0.11

**Table 4:** Relative deviation of the 17 factors in different ecosystem health regions.

Indexes Number	Ecosystem Health Levels					
	Very Good	Good	Normal	Poor	Extremely Poor	Mean
C1	−109.1	−56.4	−20.3	−16.3	−86.9	−57.8
C2	−14.7	−80.6	−150.9	−46.3	−102.8	−79.06
C3	66.1	15.8	92.4	55.4	18.8	49.7
C4	2.4	5.8	10.1	19.4	22.5	12.04
C5	−58.3	−30.8	−28.6	−20.3	−79.4	−43.48
C6	−99.3	−48.1	−140.2	−30.4	−109.5	−85.5
C7	96.3	20.5	99.1	66.3	48.9	66.22
C8	102.8	45.5	102.5	142.5	38.4	86.3
C9	114.4	37.0	40.3	150.2	67.6	81.9
C10	61.0	152.4	26.3	62.1	56.1	71.6
C11	147.5	162.8	81.2	184.7	16.7	118.6
C12	64.3	139.9	67.6	109.5	68.4	89.9
C13	29.9	70.8	64.2	194.9	40.7	80.1
C14	21.3	18.1	77.4	175.5	8.7	60.2
C15	9.7	177.2	38.1	215.9	37.5	95.7
C16	29.6	125.2	60.2	117.9	65.6	79.7
C17	15.4	87.5	58.2	180.8	23.9	73.1
